# Low Testosterone Correlates with Delayed Development in Male Orangutans

**DOI:** 10.1371/journal.pone.0047282

**Published:** 2012-10-15

**Authors:** Melissa Emery Thompson, Amy Zhou, Cheryl D. Knott

**Affiliations:** 1 Department of Anthropology, University of New Mexico, Albuquerque, New Mexico, United States of America; 2 Department of Human Evolutionary Biology, Harvard University, Cambridge, Massachusetts, United States of America; 3 University of Massachusetts Medical School, Worcester, Massachusetts, United States of America; 4 Department of Anthropology, Boston University, Boston, Massachusetts, United States of America; Max Planck Institute for Evolutionary Anthropology, Germany

## Abstract

Male orangutans (*Pongo spp.*) display an unusual characteristic for mammals in that some adult males advance quickly to full secondary sexual development while others can remain in an adolescent-like form for a decade or more past the age of sexual maturity. Remarkably little is understood about how and why differences in developmental timing occur. While fully-developed males are known to produce higher androgen levels than arrested males, the longer-term role of steroid hormones in male life history variation has not been examined. We examined variation in testosterone and cortisol production among 18 fully-developed (“flanged”) male orangutans in U.S. captive facilities. Our study revealed that while testosterone levels did not vary significantly according to current age, housing condition, and species origin, males that had undergone precocious development had higher testosterone levels than males that had experienced developmental arrest. While androgen variation had previously been viewed as a state-dependent characteristic of male developmental status, our study reveals that differences in the physiology of early and late developing males are detectable long past the developmental transition and may instead be trait-level characteristics associated with a male’s life history strategy. Further studies are needed to determine how early in life differences in testosterone levels emerge and what consequences this variation may have for male behavioral strategies.

## Introduction

**Figure 1:**
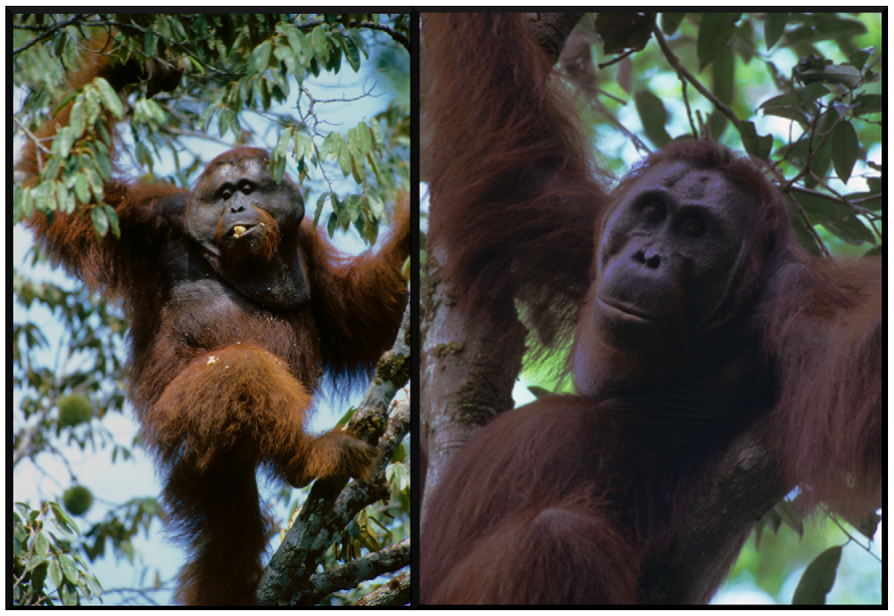
Sexual bimaturism in orangutans. Flanged (A) and unflanged (B) male orangutans from Gunung Palung National Park, Indonesia, illustrating dramatic differences in morphology. Photos by Tim Laman.

While it is common for alternative reproductive strategies to exist within a species, orangutans are unusual among mammals in that fertile, adult males occur in two distinct morphological types. Flanged males (Figure 1a) exhibit massive body size accompanied by prominent fatty cheek flanges, and a throat sac facilitating the long call vocalization of this morph [1]–[3]. Some males, although able to reproduce, remain in the adolescent-like unflanged form (Figure 1b) for 10 or more years following puberty [4], [5]. While flanged males appear to gain preferential access to fecundable females [6], it remains unclear why some males develop earlier than others, what factors trigger secondary sexual development, and what the relative fitness advantages of each morph are. Early observations from captivity suggested that development was suppressed whenever a dominant flanged male could be seen or heard [7]–[9], yet results from male pairings are inconsistent. Additionally, a survey of captive orangutans found higher levels of glucocorticoids in males undergoing secondary sexual development than those in developmental arrest, suggesting that stress from competitors was not a mechanism for delayed maturation [10]. A failure to understand the mechanisms for the male developmental transition has hindered understanding the evolution of this peculiar life history pattern.

As a general rule, testosterone and related androgenic hormones are responsible for the development of male genitalia, as well as a range of sexually-dimorphic characteristics, such as musculature and ornamental coloration. Testosterone additionally supports behaviors pursuant to male reproduction, including sexual motivation, breeding displays, and male-male competition [11]–[13]. Thus, it is not surprising that studies have reported higher testosterone levels among flanged versus unflanged males [8], [14]. The precise role of testosterone during development is still questionable, since one study in captivity found the levels of actively-developing males to be significantly higher than either arrested or fully-flanged males [14], while another found the levels of developing males to be intermediate between arrested and flanged males [8]. In neither case were young males followed to determine whether variation in testosterone levels is merely informative about current developmental status (“state-dependent”) or whether this variation might influence or predict future developmental trajectories (“trait-dependent”). Similarly, these studies do not indicate whether testosterone levels continue to change with age after development is complete. In fact, no published research explores variation in testosterone levels among orangutan males beyond the level of current developmental stage. Such research would importantly inform our understanding of alternative life history variation in orangutans by revealing whether life history strategies diverge only in age of development or encompass longer-term processes. Additionally, insights into physiological variation among flanged males may help explain the highly variable behavior patterns observed among flanged males in the wild [15], [16].

Stress can be a potent inhibitor of development, with glucocorticoids acting directly to inhibit growth hormone, thyroid hormone, and other growth factors [17], [18]. Elevated glucocorticoids can also be a mechanism for the reproductive suppression of subordinate animals [19], [20], though this can be ruled out as a mechanism for suppression in many cases [21], [22]. While it has been suggested that development in orangutan males may be suppressed directly or indirectly by the presence of dominant flanged males, a prior endocrine study found growth hormone levels of seven arrested males to be suppressed [23] without elevation of glucocorticoids [10]. Nevertheless, because glucocorticoids have interactions both with development and with the reproductive axis, they may be expected to be correlates of male life history strategy. Additionally, because some captive housing conditions may lead to chronic stress, it is important to determine whether variation in androgen production may be a correlate of variation in the stress response.

While longitudinal studies of physiology during development are needed to fully address the relationship of hormones to male life history in orangutans, the exceptionally slow developmental process in orangutans makes realizing this goal a time-consuming challenge. As we continue to gather such data, we present here an examination of variation in testosterone and glucocorticoids among fully-flanged male orangutans in relation to several important life history variables: current age, age of flange development, the presence of other flanged males in the same facility, and specific origin (*Pongo pygmaeus, P. abelii*, or hybrid). We predict that, if divergence of male life history strategies persists beyond the age of flange development, males that developed at a relatively young age will have higher testosterone than males that developed relatively late. We further predict that this difference should not be explained by variation in stress, as measured by glucocorticoid levels.

## Methods

We recruited subjects by contacting accredited U.S. captive facilities for collaboration. Thirteen zoos collected urine samples from 18 flanged male orangutans (Table 1). All subjects were captive-born and thus of known age. For 15 of the subjects, facility records provided information on developmental age, defined as the first year in which noticeable changes in secondary sexual characteristics (chiefly, body and flange size) were recorded. It should be noted that male development, once initiated, proceeds relatively rapidly and is typically completed within 1–2 years. Five of the males initiated flange development early, between the ages of 9 and 11, while 10 of the males initiated development late, between the ages of 14 and 18. Though these categories unevenly divide the males in the study, they were most appropriate because of a discrete gap in the distribution; no males of known developmental age in our sample initiated development at ages 12 or 13. For an additional 3 males of uncertain developmental age, contextual information based on developmental status at the time of transfer between facilities allowed us to determine whether they had begun development prior to age 14 (N = 1) or after age 14 (N = 2). Therefore, our final sample included 18 males that were designated as to whether their age of development was early (prior to age 14, N = 6) or late (after age 14, N = 12). All males except one were housed in social groups with at least one adult female; while mutual intolerance prevents two flanged males from living as cagemates just as it prevents direct association in the wild, some facilities maintained more than one group, meaning that males would have had some auditory, visual, and/or olfactory contact with another flanged male. Thus, we considered in our analysis whether each male was housed in the same facility as one or more flanged males. Unfortunately, record keeping was not generally adequate to determine housing conditions at the time of male secondary sexual development. In the process of collecting samples from flanged males, two collaborating zoos also submitted 25 samples from 3 males that were undergoing development at the time of sampling.

**Table 1:** Study subjects and characteristics.

Stud ID	Zoo	Species	Age at Sampling	Age at Flanging	# Samples	Mean ± S.D. Morning T(ng/mg-Cr)	Current Housed with Flanged
2431 Brunei	Brookfield	Hybrid	15	9	3	276±219	Yes
2132 Kiko	National	Hybrid	19	10	3	660	Yes
1818 Urban	Sacramento	*P. abelii*	25	11	10	146±93	No
1801 Chewbacca	Sedgwick County	*P. abelii*	26	11	8	237±198	No
2021 Rok	Little Rock	Hybrid	22	11	7	123±59	No
1886 Pongo	Brookfield	Hybrid	24	<14	6	197±71	Yes
2255 Heran	Woodland Park	Hybrid	18	14	10	52±10	Yes
1932 Mias II	Denver	*P. abelii*	23	15	6	111±31	Yes
2009 Doc	Houston	*P. pygmaeus*	24	15	10	113±34	Yes
2139 Teak	Louisville	Hybrid	20	15	10	146±58	Yes
2137 Segundo	Louisville	*P. abelii*	20	15	10	220±89	Yes
2201 Mawas	Topeka	*P. pygmaeus*	18	15	4	63±5	No
1614 Rudi	Houston	Hybrid	31	16	10	77±9	Yes
1403 Rango	Lowry Park	*P. pygmaeus*	33	16	6	38±19	No
1616 Azy	Great Ape Trust	Hybrid	29	17	18	175±45	No
2034 Butch	Miami Metro	P. abelii	21	18	6	251±148	Yes
1671 Ben	Brookfield	P. pygmaeus	28	>14	4	172±70	Yes
1504 Robin	Denver	Hybrid	30	>14	5	85±29	Yes
**Actively Developing Males**							
2718 Panji	Sedgwick County	*P. abelii*	11		8	317±197	Yes
2626 Sulango	Atlanta	*P. pygmaeus*	14		7	59±30	Yes
2259 Jantan	Atlanta	*P. abelii*	18		10	50±15	Yes

Urine samples were collected from each male on multiple days within a 1–4 week time period (Table 1). Samples were collected either by direct urination into a cup or by pipetting from a clean, dry cage floor. Samples were placed into polypropylene tubes, frozen immediately, and subsequently shipped to the laboratory on ice for analysis within 1–3 months. In other apes, urinary testosterone and cortisol are reported to have circadian patterns, decreasing from morning to afternoon [24]–[26]. We, therefore, centered our analysis on 120 samples collected prior to noon before orangutans went on display (of which >80% were collected between 7 and 9 am). Among samples collected prior to noon, there was no significant decline in either hormone among samples with a specific time recorded (log-transformed testosterone, Pearson’s r = 0.070, N = 110, p = 0.466; log-transformed cortisol, r = −0.178, N = 110, p = 0.063), which is consistent with our observations for wild orangutans (Knott and Emery Thompson, unpublished data). For six subjects sampled both in the morning and afternoon (on different days), average hormonal determinations from the two time periods were highly correlated (log-transformed testosterone, Pearson’s r = 0.932, N = 6, p = 0.007; cortisol, r = 0.904, N = 6, p = 0.013). Prior to assays, samples were deconjugated with beta-glucuronidase (*Helix pomatia*, Calbiochem, <2% aryl sulfatase activity) to recover the major urinary metabolite of testosterone [27]. Though a recent paper has questioned whether *Escherichia coli* derived enzyme might be superior to *H. pomatia* for this procedure [28], we found that the two enzyme derivations produced comparable testosterone concentrations from orangutan urine (log-transformed, Pearson’s r = 0.709, N = 35, p<0.001; *E. coli* VII-A, Sigma Aldrich). After drying down and reconstituting in phosphate-buffered saline, samples were assayed for immunoreactive testosterone and cortisol metabolites using enzyme-immunoassay reagents and protocols provided by the Clinical Endocrinology Laboratory at UC Davis [29].

The testosterone antibody employed (R156/7, U.C. Davis) has demonstrated a high cross-reactivity with only one exclusive metabolite of testosterone, 5alpha-dihydrotestosterone (DHT, 57%), and low cross-reactivity with the adrenal androgen androstenedione (0.3%, C. Munro personal communication). Cross-reactivities with other steroids were minimal (≤0.04). Assay accuracy was assessed by the recovery of testosterone in an orangutan urine sample added to the standard curve doses. Recoveries averaged 121±14 (SD)% (N = 7). This suggests that our assay systematically overestimates the concentrations of testosterone in orangutan urine. However, variation in recovery did not covary with the dosage of standard and could not be attributable to differences in sample matrix as both standard and sample were brought up in the same buffer. We also tested the parallelism of binding curves generated from serial dilutions of testosterone standard and an extracted orangutan urine sample. The resulting regressions were not significantly different (standard y = −0.380x +1.512, sample: y = −0.337x +1.322; t = 0.932, df = 10, p = 0.373). Interassay CVs were 12.4% for low samples and 15.4% for high sample (N = 12), and intrassay CVs for duplicates (N = 161) averaged 7.7%. Sensitivity of the assay was approximately 16 pg/ml.

The cortisol antibody (R4866, U.C. Davis) used in our assay cross-reacts to a limited degree with cortisone (5%), while all other cross-reactivities with natural steroids are <1%. Recoveries of cortisol in orangutan urine averaged 90.5±8.7% (N = 7). Parallel curves were obtained from diluted standard and urine samples (standard y = −0.115x +0.979, sample: y = −0.138x +0.978; t = −0.392, df = 9, p = 0.704). Interassay CVs were 10.9% for low samples and 13.6% for high samples (N = 15), and intraassay CVs averaged 7.1% (N = 161). Sensitivity of the assay was approximately 16 pg/ml. Steroid concentrations were indexed to creatinine using the Jaffe reaction [30] and log-transformed prior to analysis.

Statistical analyses were performed using PASW 18.0. Generalized Linear Models included two discrete factors (age of development early/late and presence/absence of flanged male in the same facility) and one continuous covariate (age), with each male’s mean immunoreactive testosterone or cortisol as the response variable. Non-significant variables were sequentially eliminated until the model contained only predictors with p-values <0.05. Parameter and probability estimates for excluded predictors, as presented in the tables, are calculated by adding each back, one at a time, to the final model. We tested for possible significant interaction terms, but found none. We also verified that the models produced normally-distributed and uncorrelated residuals and that significant predictors exhibited homogeneity of variance. Species origin does not qualitatively affect our results if included in such models, but we did not include it because there was an empty category (all Bornean were late maturers). Effects of species were examined in separate one-way ANOVAs.

Non-invasive sample collection was conducted with approvals by the Animal Care and Use Committees at Harvard University (protocol 95-04) and the University of New Mexico (protocol 11-100726-MCC). Samples were collected without direct contact with the animals and with minimal disturbance to their daily routine.

## Results

**Figure 2:**
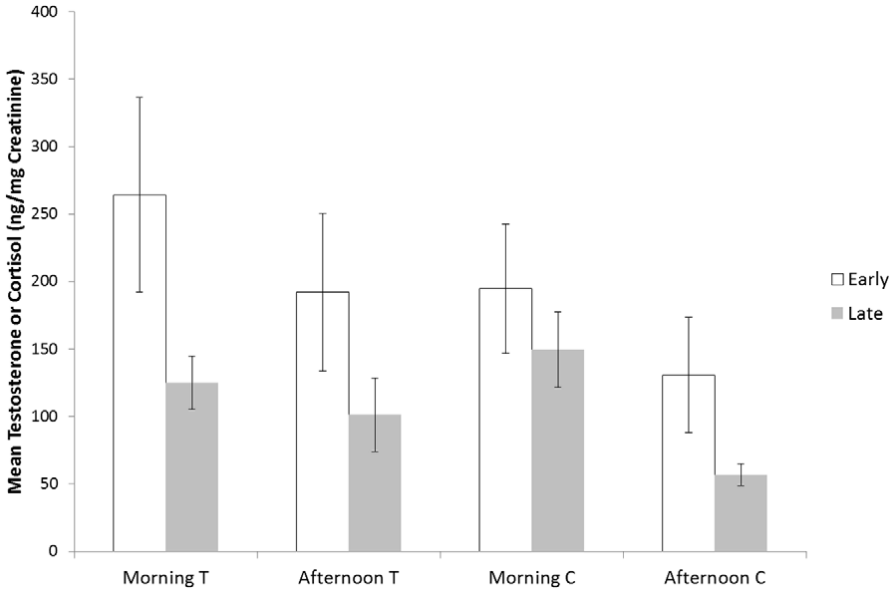
Boxplot of urinary testosterone and cortisol in captive male orangutan subjects according to categorical age of maturation: early (flanging before age 14) or late (after age 14). Morning samples were obtained from 18 subjects and comprised the dataset for statistical analysis; afternoon samples were available for only 6 males but corroborate the differences observed in the morning. Plots indicate median (horizontal line), 25^th^ and 75^th^ percentile (box), minimum and maximum excluding outliers (whiskers), and outliers (circles).

While neither age nor presence of other flanged males was a significant predictor of urinary testosterone levels, age of flange development was (Table 2). Males that completed their development before age 14 had higher testosterone levels as adults than did males that completed development after that age (Figure 2). One male with unusually high testosterone levels and a single morning sample did not bias the results, as the model was qualitatively the same and the effect of developmental timing was statistically significant with his exclusion (Estimate = 0.240, df = 1, p = 0.039).

**Table 2:** Results of Generalized Linear Models for Urinary Testosterone in 18 Flanged Male Orangutans.

Variable	Estimate	Std. Error	Df	p-value
Intercept	2.033	0.069	1	<0.001
Early/Late Development	0.325	0.120	1	0.007
*Excluded Variables*				
Age	−0.015	0.011	1	0.201
Other Flanged	0.137	0.122	1	0.261

Urinary cortisol levels of males were not correlated with their testosterone levels (Pearson’s r = 0.233, N = 21, p = 0.310). Cortisol levels did not vary significantly between early- and late-maturing males, between species, between males housed in proximity with other flanged males and those not, nor with male age (Table 3).

**Table 3:** Results of Generalized Linear Models for Urinary Cortisol in 18 Flanged Male Orangutans.

Variable	Estimate	Std. Error	Df	p-value
Intercept	2.143	0.059	1	<0.001
*Excluded Variables*				
Age	−0.017	0.011	1	0.135
Early/Late Development	0.131	0.121	1	0.281
Other Flanged	0.002	0.121	1	0.990

Because all four Bornean orangutans in our sample matured late, we tested whether there was a species effect on either testosterone or cortisol and found no evidence of such an effect in either the full dataset (ANOVA, testosterone: F = 1.833, df = 2,15, p = 0.194; cortisol: F = 0.378, df = 2,15, p = 0.691) or in the subset of subjects that matured late (ANOVA, testosterone: F = 1.975, df = 2,9, p = 0.195; cortisol: F = 0.205, df = 2,9, p = 0.818).

## Discussion

In this first analysis of variation in testosterone among flanged male orangutans, we found that males that had completed their secondary sexual development at a young age maintained higher testosterone levels than those with delayed maturation. This is the first evidence that testosterone levels in male orangutans are linked not only with developmental status, but with developmental timing. This extends our understanding of a very rare form of developmental variation by demonstrating persistent differences in the steroid physiology of males with divergent life history strategies. The fact that developmentally delayed males maintain low levels of androgen production many years after transitioning to a flanged status suggests that they may continue to pursue differences in behavioral strategies when compared to males with early development, a possibility which has not yet been raised in the literature on this species. Indeed, if it is the case that all males eventually complete secondary sexual development, categorization of behavior according to current status may have blurred the lines between males pursuing dramatically different life history strategies.

One possible alternative explanation for the link between testosterone production and developmental age is that some males may have experienced persistent stress in their environments, simultaneously delaying development and suppressing testosterone. Indeed, the prevailing hypothesis for arrested development in orangutans is that male secondary sexual development is delayed and subsequently triggered in response to external stimuli, such as visual or auditory cues to the presence of other developed males [7], [9]. No such triggers have been consistently linked with developmental timing, and a prior study reported that arrested males actually had lower stress hormone production than flanging males [10]. Our results found no difference in stress hormone levels between early and late developing males. Because flanged males compete intensely with one another but are relatively tolerant of unflanged males, it has been suggested that delayed development could actually serve as a mechanism for stress avoidance in the face of intense competition [10]. If this is the case, then we would expect stress hormone levels to equalize after flanging, consistent with our findings. Nevertheless, the stress- avoidance hypothesis suggests that there should be stress responses to male competitors, and we did not find that the presence of other flanged males in the same facility produced an effect on either testosterone or cortisol levels of the subjects.

Neither current age nor species origin was a significant predictor of testosterone or cortisol in our sample. Age declines in testosterone are detectable in some human populations [31], as well as some non-human primates (e.g., baboons [32], but not muriquis [33]). Our data showed a trend in this direction, but given a large amount of individual variability, age trends are difficult to discern in small samples. The solitary nature of orangutan males makes it difficult to acquire a sufficiently large sample from either the wild or captivity to gain sufficient power for such a test. Additionally, the eldest males in our sample were in their mid-30s, whereas orangutans can live a decade or two longer [34], [35]. There is a debate in the literature as to whether the Bornean and Sumatran species of orangutans maintain evolved differences in life history trajectories [36]. While some authors have argued for slower life histories for Sumatran orangutans in the wild based on small samples of interbirth intervals [35], demographic statistics for the two species converge in captivity when ecological conditions are stable [34]. Thus, while it has been suggested that developmental arrest might be more common or more prolonged among Sumatran orangutans, where the relative proportion of unflanged males is higher [37], this was not apparent in our captive sample: all four Bornean males were late-maturers, and species origin did not modify the relationship between maturation timing and hormonal parameters. However, the majority of individuals in our sample were hybrids.

Our results cannot currently distinguish between two alternative interpretations, that young male orangutans that produce high levels of testosterone initiate secondary sexual development earlier than those with low levels, or that males that developed early attain higher testosterone levels only after flanging. Distinguishing these alternatives will require monitoring young male orangutans throughout the long juvenile and adolescent periods. Acquiring such a sample will require many years of continuing research, and is an objective we are actively pursuing. However, in acquiring the sample for the present study, we incidentally received samples from 3 males that were beginning flange development. Among them, one was developing at a young age (8 yrs) and had testosterone levels more than five times higher than the two males that were developing at late ages (14 and 18 yrs). Other studies have identified predictive effects of testosterone on future status. Testosterone levels of chacma baboons over a 1-yr study period were better predictors of future than current rank and mating activity [38]. In the closest primate analog to male bimaturism in orangutans, male mandrills commonly experience delayed reproductive development. Among adolescent male mandrills, there is already considerable individual variation in testosterone levels, which correlates with individual differences in secondary sexual development, testicular volume, body size, and dominance and with early attainment of adult weight [39], [40]. Evidence of similar effects in orangutans would modify hypotheses about developmental timing in this species to focus less on the role of external factors and more on the role of intrinsic individual variation.

It is not currently known what consequences the observed variation in testosterone would have for orangutans on different life history trajectories. Elevated testosterone is generally indicative of behavioral and physiological allocations to mating effort, including competition with other males [12], [13]. Encounters among flanged male orangutans are invariably aggressive and entail significant risk of injury or death [1], [16], [41]. Therefore, it is possible that testosterone variation influences the willingness of males to provoke an encounter with another male. Flanged males differ amongst one another in the use of sexually coercive aggression with females. Ranging patterns of flanged males also vary, with some establishing home ranges and others pursuing transient strategies [4], [42]. Physiological correlates of these behaviors are not yet known, yet either might reflect persistence of high or low risk strategies past the age of development. Finally, though empirical evidence from mammals is scarce, testosterone, either directly or through its diversion of energy to mating effort, is theoretically predicted to compromise immune function and other investments in somatic maintenance [43], [44]. Just as alternative developmental trajectories in orangutans have been theorized to optimize the costs of benefits of large body size and secondary sexual adornments [1], [14], [45], [46], males are predicted to balance the costs and benefits of elevated testosterone in pursuit of a life history strategy.

In conclusion, we report that male orangutans that undergo arrested secondary sexual development maintain lower levels of androgen production, even many years following the developmental transition, than do males that develop at a young age. These differences cannot be explained by differences in housing conditions, physiological stress, or species origin, and are independent of the ages of the subject. We conclude that testosterone production is not only elevated in orangutans as a state-dependent consequence of becoming a flanged male, but is also elevated as a persistent trait of males that adopt an early maturation strategy. Thus, a full understanding of sexual bimaturism as a life history strategy in orangutans entails a longer-term perspective than that which has previously been applied and requires investigation of how developmental timing correlates with differences in behavior and physical condition after, and perhaps before, the developmental transition.
